# Demographic and Clinical Characteristics of Military Service Members Hospitalized Following a Suicide Attempt versus Suicide Ideation

**DOI:** 10.3390/ijerph16183274

**Published:** 2019-09-06

**Authors:** Brianne J. George, Sissi Ribeiro, Su Yeon Lee-Tauler, Allison E. Bond, Kanchana U. Perera, Geoffrey Grammer, Jennifer Weaver, Marjan Ghahramanlou-Holloway

**Affiliations:** 1 Independent researcher, Frisco, TX 75034, USA; 2 The Department of Psychology, George Mason University, Fairfax, VA 22030, USA; 3 The Department of Medical & Clinical Psychology, Suicide Care, Prevention, and Research Initiative; The Uniformed Services University of the Health Sciences, Bethesda, MD 20814, USA (S.Y.L.-T.) (K.U.P.); 4 The Department of Psychology, The University of Southern Mississippi, Hattiesburg, MS 39406, USA; 5 Greenbook TMS NeuroHealth Centers, McLean, VA 22102, USA; 6 Fort Belvoir Community Hospital, Fort Belvoir, VA 22060, USA

**Keywords:** military, suicide attempt, suicide ideation, psychiatric inpatients, clinical characteristics

## Abstract

Psychiatric hospitalization for a suicide attempt (SA), rather than suicide ideation (SI) alone, is a stronger risk indicator for eventual suicide death. Yet, little is known about demographic and clinical characteristics differentiating those admitted for SA versus SI. Understanding these differences has implications for assessment and treatment. A retrospective review of electronic medical records (EMRs) was performed on service members (*n* = 955) admitted for SA or SI at the Walter Reed Army Medical Center between 2001–2006. Service members hospitalized for SA were younger compared to those hospitalized for SI. The proportion of women admitted for SA was significantly higher than those admitted for SI whereas their male counterparts showed the opposite pattern. Patients admitted for SA, versus SI, had significantly higher prevalence of adjustment disorder with mixed disturbance of emotion and conduct (MDEC), personality disorder not otherwise specified (PDNOS), and borderline personality disorder (BPD). Patients admitted for SI had significantly higher prevalence of adjustment disorder with depressed mood and deferred Axis II diagnosis, compared to those admitted for SA. There were no significant between-group differences in the average or median number of documented prior suicide attempts. Findings highlight the need for more standardized assessment, diagnostic decision-making, and documentation practices for all patients.

## 1. Introduction

Suicide is a major public health concern. According to the World Health Organization (WHO), global deaths by suicide total nearly 793,000 individuals annually [1]. In the United States (U.S.), approximately 47,173 individuals died by suicide in 2017 [2]. Suicide is the tenth leading cause of death in the U.S. [3] and currently the leading cause of death among U.S. military personnel [4]. In 2016, 21 suicide deaths occurred per 100,000 active duty service members, and this suicide mortality rate has remained steady since 2011 [5]. While the Department of Defense (DoD) historically reported lower suicide rates among military service members compared to civilians [6], age- and sex-adjusted suicide rates across military and civilian populations have become comparable since 2009 [7].

Individuals having experienced a suicide attempt (SA) or suicide ideation (SI) are at elevated risk of suicide death [8]. Based on the WHO’s multi-national survey, lifetime prevalence of SA and SI worldwide are estimated at 2.7% and 9.2%, respectively [9]. One-third of these individuals with ideation ever make a suicide attempt, and two thirds of these attempts occur within the first year of the onset of suicide ideation. Compared to civilians, lifetime prevalence of SA and SI appear to be twice as higher among U.S. military service members—5.1% and 18.1%, respectively [10].

Less is known about demographic and clinical factors differentiating psychiatric patients admitted due to SA compared to those admitted for SI [11]. While the majority of individuals with SI do not attempt suicide, SA is one of the most robust clinical indicators of eventual death by suicide [12]. Understanding factors specifically associated with SA (i.e., behaviors) versus SI alone (i.e., thinking) is expected to improve both assessment and treatment approaches for individuals at risk of suicide. For example, based on a systematic review, a history of sexual abuse as well as the diagnosis of post-traumatic stress disorder (PTSD) are reported as more common among individuals with suicide attempts compared to individuals with suicide ideation [11]. Moreover, recent studies have suggested that some psychosocial factors, such as stressful life events and emotional abuse, are more common among individuals with SA compared to SI [13,14]. As stated earlier, further efforts to distinguish individuals who have attempted suicide from individuals with SI can improve targeted suicide prevention and intervention efforts [15]. To date, studies on clinical characteristics of patients with SA and SI status have been conducted among adolescents admitted to the emergency department, observation stays, and inpatient psychiatric units [16]; the studies have examined demographic differences such as age, sex, and race and/or ethnicity. Describing the demographic and clinical characteristics across patients with SA and SI status remains a gap in inpatient adult psychiatric research, as well as among military service members.

Determining who is at risk for experiencing suicide ideation compared to attempting suicide is an important task for suicide prevention researchers. A recent study found that little is known about the factors that increase one’s risk for suicide [17]. One way to determine suicide risk is differentiating the demographic and clinical characteristics between individuals presenting suicidal thoughts only, compared to individuals who have recently attempted suicide. The purpose of this study was to describe the demographic and clinical characteristics of active duty service members admitted to a psychiatric inpatient unit due to either: (1) SA; or (2) the presence of SI warranting acute care. Please note that those in the SI group may have included patients with lifetime suicide attempts; however, for the purposes of this study, we only examined the SA versus SI differences based on documented reasons for the index psychiatric hospitalization. This study was exploratory in nature. However, based on the annual report on the 2017 Department of Defense suicide events [18] and published literature [19,20,21,22], we expected that female gender, lower military rank, adjustment disorder, mood disorder, personality disorders, and PTSD may be more prevalent among individuals who are psychiatrically admitted for suicide attempts compared to those admitted for suicide ideation.

## 2. Materials and Methods

A retrospective chart review was performed. The data came from a subset of the medical data used for a larger study which aimed at characterizing the differences between patients admitted for SA, SI, or non-suicide related events.

Patients admitted to the inpatient psychiatric unit from January 2001 to December 2006 at the Walter Reed Army Medical Center (WRAMC) were identified using the Essentris electronic medical record (EMR) system [23]. The inclusion criteria were: (1) inpatient psychiatric hospitalization due to SA or SI at the time of admission; and (2) status of active-duty, reserve, or National Guard at the time of admission. A total of 1003 cases met the inclusion criteria. The categorization into either SA or SI groups was based on EMR documented reason for admission as opposed to lifetime history of non-fatal self-injurious suicidal thoughts and/or behaviors. The operational definition for SA status group membership was an individual specifically hospitalized for a suicide attempt; conversely, the operational definition of SI status group membership was an individual specifically hospitalized for SI, regardless of prior suicide attempt history. Duplicate records were excluded from the total number, resulting in a final sample of 955 unique cases, which included 421 SA cases and 534 SI cases.

A coding manual was developed in consultation with a number of suicidology and military mental health care experts, which served as the basis for the electronic medical record—coding template (EMR-CT). Clinical psychology doctoral candidates served as coders, and their inter-rater reliability was calculated across 8 items, where the kappa coefficient ranged from 0.5 (history of adulthood negative life events) to a kappa of greater than 0.7 for the remaining 7 variables [24,25]. Information regarding demographic and clinical characteristics were captured in the EMR-CT. No Health and Insurance Probability and Accountability Act (HIPAA) identifiers were recorded on the patient’s coding forms. A unique study identification number was assigned to each record included.

Descriptive statistics were calculated to provide demographic (i.e., sex, age, race, and marital status), military service (i.e., branch and pay grade), and clinical characteristics (i.e., Axis I and Axis II diagnoses) for the entire sample. For categorical variables, percentages were calculated.

Chi-squared (χ^2^) analyses were conducted to assess the differences in demographic and clinical variables across SA and SI status. For variables with fewer than 5 records in a given cell, Fisher’s exact statistics were calculated. Independent sample t-tests were conducted for continuous variables, such as age or length of hospitalization.

Assuming a small (0.2) effect size [24], the estimated minimum sample size necessary for a two-group χ^2^ test with a 0.05 two-sided significance level to have 80% power to detect differences in each demographic and clinical characteristic was calculated at 197 records, using G*Power (Heinrich Heine University, Dusseldorf, Germany), version 3 for Macintosh [26]. Therefore, the sample (*n* = 955) was adequately powered for these analyses.

## 3. Results

### 3.1. Overall Sample Charecteristics

The sample comprised of 955 military service members psychiatrically hospitalized for inpatient care at WRAMC due to SA or SI between 2001 and 2006. Table 1 shows the demographic and military characteristics of the sample based on information recorded in the EMR. The mean age of patients in the sample was 26.3 (*SD* = 8.0). Men comprised 68.8% (*n* = 657) of the sample and women 31.2% (*n* = 298). The majority of the patients in the sample were White (64.4%), followed by African American (20.9%), Hispanic (5.4%), and 9.2% of other races. At the time of hospitalization, the majority of the patients in the sample were single (48.6%), followed by 35.9% being married, and 15.5% of patients were divorced, separated or widowed.

The military branches represented were the Army (69.2%), Air Force (13.4%), Navy (9.8%), Marine Corps (6.3%), and Coast Guard (1.3%). The majority of those hospitalized were junior enlisted (E-1–E-4; 69.7%), followed by junior non-commissioned officers (E-5–E-6; 19.5%). The remaining rank categories consisted of senior non-commissioned officers (E-7–E-9; 4.5%), company grade officers (O-1–O-3; 4.2%), and field grade officers (O-4–O-6; 1.9%).

Common primary psychiatric diagnoses were: adjustment disorder with mixed disturbance of emotion and conduct (MDEC) (26.4%), major depressive disorder (25.2%), adjustment disorder with depressed mood (10.9%), PTSD (8.8%), dysthymic disorder (7.4%) and bipolar disorder (4.3%). Prevalence of alcohol abuse and dependence were 8.5% and 11.5%, respectively. Furthermore, about a quarter (25.1%) of the sample received an Axis II personality disorder diagnosis after admission evaluations and the remainder of the sample had either no diagnosis annotated (42.0%) or a deferred diagnosis (12.0%) on Axis II.

### 3.2. Demographic Characteristics across SA and SI Groups

Of the 955 total records, 421 patients (44.1%) were admitted due to a suicide attempt, whereas 534 (55.9%) patients were admitted due to suicide ideation alone. Individuals with SA were significantly younger (*M* = 25.6) than individuals with SI (*M* = 26.3). For both males and females, the age distribution negatively skewed towards a greater frequency of younger adults within the sample. A higher percentage of male service members underwent SI (74.2%) compared to SA (62.0%); while female service members had higher percentage of SA (38.0%) compared to SI (25.8%). No significant between-group differences were noted on race, marital status, and military service branch and rank.

### 3.3. Clinical Charecteristics across SA and SI Groups

Table 2 shows the clinical characteristics of the sample across SA and SI groups. Chi-squared analyses indicated that adjustment disorder with MDEC was diagnosed in a significantly higher percentage of patients in the SA group compared to those in the SI group, (*p* < 0.001). Conversely, there was a higher percentage of adjustment disorder with depressed mood diagnoses for patients with SI compared to patients with SA (*p* = 0.039). None of the other Axis I diagnoses were notably different between the SA and SI groups. A significantly greater percentage of SA patients, compared to the SI patients, received a personality disorder diagnosis not otherwise specified (PDNOS; *p* = 0.010), and a borderline personality disorder diagnosis (BPD; *p* = 0.022).

Approximately 4 out of 10 patients in both the SA (40.1%) and SI (43.3%) groups did not receive a psychiatric diagnosis on Axis II. However, the EMR review showed that there was a significantly greater percentage of deferred diagnosis on Axis II for those in the SI group (14.0%), compared with the SA group (9.5%) (*p* = 0.032).

In terms of lifetime histories of suicide ideation and attempts, approximately 37% (*n* = 353) of the total sample had a documented history of at least one suicide attempt prior to the documented hospitalization. Of those who had a documented history of at least one prior suicide attempt, there were 324 individuals who reported a discrete number of suicide attempts (versus those whose records indicated “multiple” suicide attempts). For those patients who had one or more documented prior suicide attempts (i.e., occurring prior to hospitalization date included in the EMR), a Mann-Whitney test indicated that number of prior suicide attempts did not differ between individuals hospitalized for SA (median = 1, range 1–8) compared to individuals hospitalized for SI (median = 1, range = 1–6) groups, *U* = 12,827.0, *p* = 0.708. An independent samples t-test indicated no significant between-group differences in the average number of documented prior suicide attempts, *t*(322) = −0.071, *p* = 0.937. Furthermore, analyses on the categorical number of prior suicide attempts (i.e., none, one, two or more) indicated no significant between-group differences. A significantly greater number of SI cases, compared to SA cases, were missing the prior suicide attempt information, χ^2^ (1, *n* = 955) = 15.796, *p* < 0.0001. 4% of the SA group records versus 11% of the SI group records did not contain documentation on prior lifetime SA.

## 4. Discussion

This study shows that younger and female military personnel were more likely to be psychiatrically hospitalized for suicide attempts compared to suicidal ideation. Military personnel admitted for a suicide attempt compared to suicidal ideation were more likely to receive psychiatric diagnoses reflective of conduct problems (e.g., adjustment disorder with MDEC) and personality pathology (e.g., BPD and PDNOS). This study presents a snapshot of how EMR could be used to assess patient characteristics based on medical providers’ documentation for patients admitted due to suicidal thoughts and/or behaviors.

While junior enlisted members (lower military rank than officers) appeared to be equally likely to be hospitalized for SA or SI, service members hospitalized due to SA were younger compared to individuals hospitalized for SI. Younger service members, regardless of their military rank, who are expected to be earlier in their career, were more prone to be hospitalized for suicide attempt [27].

The proportion of women in the SA group was significantly greater than those in the SI group, whereas their male counterparts showed the opposite pattern. For the overall sample (regardless of suicide status psychiatric admission), females were overrepresented by about one-third of the sample when compared to the general military population, as the average representation of women in the U.S. military is about 16.2% [18]. This higher proportion of females in an inpatient psychiatric setting compared to the overall makeup of the military total forces is in line with the 2017 Department of Defense Suicide Event Report (DoDSER) showing that female service members accounted for 30.1% of suicide attempts. Based on a crude comparison, it appears that proportion of female suicide attempt in the military (30.1%) is comparable to the proportion of female psychiatric inpatients admitted due to suicide attempt (38%) [18], suggesting that female service members with recent suicide attempts are accessing acute care. However, carefully designed studies are necessary to ascertain if female service members with suicidal behaviors are receiving adequate care. These findings are in line with epidemiological data documented previously in a sample of 87,257 female and 70,570 male adults, illustrating a greater rate of SA hospitalizations in the female sample [28], but empirical information on rates of SA versus SI hospitalizations remains scarce.

The majority of the sample had at least one Axis I diagnosis, most commonly a mood or an adjustment disorder, followed by a substance-related and an anxiety disorder. In 2012, these four diagnoses were among the top five conditions accounting for over half the total Department of Defense hospital bed days [29]. Within the U.S., the most common diagnoses for individuals hospitalized in 2011 included mood disorders, dementia-related and cognitive disorders, anxiety disorders, and substance-related disorders [30]. In this study, no significant group differences were found in terms of the categories of mood, PTSD, or substance use disorders. If the SA group is conceptualized as a subset of patients with this set of psychopathologies [31], our analyses do not support this expectation.

Patients in the SA group had significantly higher prevalence of adjustment disorder with MDEC than those in the SI group, whereas patients in the SI group had a significantly higher prevalence of adjustment disorder with depressed mood. For clarification, adjustment disorder with the specifier of MDEC captures the disruption of the individual due to anxiety, depression, and the distress and behavior changes due to stressful life events while the specifier of depressed mood describes symptoms like those of depression [32]. These differential specifiers used for adjustment disorder may reflect the true clinical differences or clinical bias at the time of suicide-related admission. While we cannot ascertain how medical providers made the clinical decision to diagnose MDEC or depressed mood, it is also possible that MDEC was diagnosed in individuals who were hospitalized due to a suicidal behavior. There is a possibility that clinical bias was involved in the diagnosis of MDEC for individuals hospitalized following SA, as opposed to primary mood-related specifier documented on individuals admitted for SI only.

With regards to personality disorder diagnoses, PDNOS and BPD were more prevalent in the SA group. Additionally, those in the SI group had a significantly greater proportion of deferred Axis II diagnoses compared to the SA group. The diagnosis of a personality disorder is a sensitive issue, especially within the U.S. military population. Personality disorder diagnoses are listed as an unsuitable condition for military service, especially if the disorder impacts a person’s functioning or ability to carry out the military mission [33]. The higher percentage of deferred Axis II diagnoses within the SI group may be in part due to mental health providers being cautious with giving a potentially career-effecting diagnosis when a military patient has not attempted suicide.

PDNOS is not as specifically defined as other personality disorders, which leads to lack of consistency in its diagnosis [34]. PDNOS could be used as a “catch-all” for individuals who may not meet the criteria for a specific personality disorder (PD) [34]. Because personality disorders are not seen as a disease or injury, they render a service member unsuitable for military service and are not eligible for post-separation compensation. Unsurprisingly, these diagnoses were more frequent among individuals with SA; individuals with BPD are at an increased risk of SA, especially when present with Axis I disorders [35]. Such comorbid disorders include major depressive disorder (MDD), PTSD, and alcohol-related disorders; further history of traumatic events increases suicide risk within this population. BPD is also found to associate with multiple SAs among military service members who were hospitalized for an acute suicide-related event [36].

The personality disorder diagnoses presented here were all given in the context of admission to a psychiatric inpatient setting—that is, within the first 48 h of admission. There was no clear documentation within the EMR about what formal and/or informal assessments were conducted to reach this career-impacting diagnosis. A safe assumption is that that diagnoses were made based on a clinical interview. Finally, our findings indicated that the two suicide status groups did not differ significantly in terms of their mean or median number of previous suicide attempts. We want to interpret this non-difference with caution, however, since a significantly greater number of SI cases were missing the prior suicide attempt information compared with the SA cases. The fact that a significantly higher proportion of individuals hospitalized for SI were missing prior suicide attempt history compared to individuals hospitalized for SA suggests that medical providers may be more likely to overlook documentation of prior suicide-related events among patients being admitted for SI. We suggest that medical providers should have a standard approach to document patients’ prior suicide attempt histories irrespective of their admission status.

A clinical as well statistically significant finding was that individuals in the SI group showed greater percentage of unknown histories of suicide attempts compared with the SA group. Mental health providers are constantly educated about the importance of asking about and documenting information about prior suicide attempt behavior of patients at risk for suicide. Patients psychiatrically hospitalized for suicide ideation and attempts are a highly vulnerable group. Medical documentation must reflect this type of information and its absence indicates either that the provider did not ask about the information (which reflects problematic care) or did not document the information appropriately (which reflects problematic record-keeping).

The scientific literature indicates that individuals who presented for medical care were not often asked about suicide ideation, and up to 2% of those with suicide ideation had a suicide plan [37]. This is significant because patients in the SI group had the same mean number of suicide attempts when the number was known. Despite having suicide ideation strong enough to warrant psychiatric hospitalization, an unknown lifetime suicide attempt history was more frequently noted in the records of those with suicide ideation. Understanding that patients may not spontaneously disclose their suicide history is key in facilitating a thorough risk assessment, as we know that risk cannot be ascertained without asking a person if they are suicidal [38].

### 4.1. Limitations and Strengths

As per our study design, the analyses of demographic and clinical characteristics across SA and SI military inpatients were explorative without inference to causal relationship. The lack of standardization within the EMR created two important study limitations. First, while the study’s coding template was standardized with categorical options, some EMRs presented conflicting or ambiguous information, which increased uncertainties in categorization. Particularly, while SA and SI are broad categories for the reasons for psychiatric hospitalization, we were unable to assess the level of suicide risk severity. Additionally, psychometrically sound assessment measures were not included in the EMR and the data came entirely from the record of mental health providers’ diagnoses. Another study limitation is related to generalizability, as the sample characteristics are not representative of the wider military population.

As for strengths, this study provides insights into the characteristics of service members requiring psychiatric hospitalization due to recent SA or SI. To date, the Department of Defense has paid primarily close attention to the characteristics of military personnel who die by suicide, as reflected by the DoD Suicide Event Report publications. Yet minimal data exists on those who attempt suicide, and even less exists on those with suicide ideation requiring psychiatric hospitalization. Moreover, given the large sample size, adequate power was available to generate study conclusions.

### 4.2. Recommendations and Implications

In terms of research, a longitudinal examination of patients psychiatrically admitted for SA versus SI could provide useful information about suicide risk trajectories over time. Given the absence of comprehensive symptom-level data in EMR, policy initiatives may include further standardization of documentation practices on military inpatient psychiatric units. For example, using a standardized checklist or assessment tool (e.g., the Columbia Suicide Severity Rating Scale) [39] for suicide ideation and behaviors may provide a comprehensive plan of action for admitted patients and also guide the decision-making process for discharges from the inpatient unit. Standardized assessment and documentation practices will allow for a more enhanced delivery of care, which can be tailored to the unique treatment needs of each admitted patient. A more reliable and comprehensive EMR would improve subsequent care as well as the accuracy of research and compliance reviews.

## 5. Conclusions

This study examined the demographic and clinical characteristics of U.S. active duty service members admitted for psychiatric inpatient care for a suicide attempt versus suicide ideation severe enough to warrant hospitalization. The two groups (SA and SI) did not differ in their documented prior suicide attempts. Findings based on a retrospective chart review indicate that those in the SA group were notably younger compared with those in the SI group; this finding is supported by published civilian epidemiological data [9]. The proportion of women was higher in the SA group, and this highlights that while military males have higher rates of suicide deaths in the military, their female counterparts require much attention in relation to non-fatal suicidal self-directed violence. Service members admitted following an SA, compared with SI, had a significantly higher prevalence of adjustment disorder with MDEC, PDNOS, and BPD, whereas those admitted for SI had a significantly higher prevalence of adjustment disorder with depressed mood and a deferred Axis II diagnosis. We conclude that more standardized assessment, diagnostic decision-making, and documentation practices for inpatient psychiatric care are needed. A one-size-fits all approach may not be the most suitable for those admitted due to a recent suicide attempt versus those admitted for suicide ideation, given some of the differences highlighted in our research.

## Figures and Tables

**Table 1:** Demographic characteristics across military psychiatric inpatients hospitalized for suicide attempt versus suicide ideation (*n* = 955).

Characteristic	Total (*n* = 955)		Suicide Ideation (*n* = 534)		Suicide Attempt (*n* = 421)		*t*-Statistic	
	*M*	*SD* (Range)	*M*	*SD* (Range)	*M*	*SD* (Range)	*t (df)*	*p*
Age (in years)	26.3	8.0 (17–60)	26.8	8.5 (17–60)	25.6	7.2 (17–59)	−2.312 (953)	0.021
	***n***	**%**	***n***	**%**	***n***	**%**	**χ^2^*(df)***	***p value***
Gender				
Men	657	68.8	396	74.2	261	62.0	16.220 (1)	<0.001
Women	298	31.2	138	25.8	160	38.0		
Race					
White	615	64.4	344	64.4	271	64.4	0.724 (3)	0.867
African American	200	20.9	108	20.2	92	21.9		
Hispanic	52	5.4	30	5.6	22	5.2		
Other	88	9.2	52	9.7	36	8.6		
Marital Status					
Single	464	48.6	256	47.9	208	49.4	0.605 (2)	0.739
Married	343	35.9	191	35.8	152	36.1		
Divorced/Separated/Widowed	148	15.5	87	16.3	61	14.5		
Service Branch					
Army	661	69.2	365	68.3	296	70.3	1.078 (4)	0.898
Air Force	128	13.4	76	14.2	52	12.4		
Navy	94	9.8	52	9.7	42	10.0		
Marine Corps	60	6.3	35	6.6	25	5.9		
Coast Guard	12	1.3	6	1.1	6	1.4		
Rank ^^^					
E-1–E-4	666	69.7	365	68.4	301	71.5	5.366 (4)	0.252
E-5–E-6	186	19.5	102	19.1	84	20.0		
E-7–E-9	43	4.5	28	5.2	15	3.6		
O-1–O-3	40	4.2	28	5.2	12	2.9		
O-4–O-6	18	1.9	9	1.7	9	2.1		

Note: ^^^ Two participants were excluded due to low cell counts; *M*: mean; *SD*: standard deviation; *df*: degrees of freedom.

**Table 2:** Clinical charecteristics across military psychiatric inpatients hospitalzied for suicide attempt versus suicide ideation (*n* = 955).

Clinical Characteristics	Total (*n* = 955)		Suicide Ideation (*n* = 534)		Suicide Attempt (*n* = 421)		*t*-Statistic	
	*n*	%	*n*	%	*n*	%	χ^2^ (*df*)	*p-Value*
Axis I Primary Diagnoses					
Adjustment Disorder with Depressed Mood	104	10.9	68	12.7	36	8.6	4.245 (1)	0.039
Adjustment Disorder with Mixed Disturbance of Emotion and Conduct	252	26.4	117	21.9	135	32.1	12.501 (1)	<0.001
Alcohol Abuse	81	8.5	44	8.2	37	8.8	0.091 (1)	0.762
Alcohol Dependence	110	11.5	63	11.8	47	11.2	0.093 (1)	0.761
Bipolar Disorder	41	4.3	24	4.5	17	4.0	0.119 (1)	0.730
Dysthymic Disorder	71	7.4	39	7.3	32	7.6	0.030 (1)	0.862
Major Depressive Disorder	241	25.2	147	27.5	94	22.3	3.374 (1)	0.066
Posttraumatic Stress Disorder	84	8.8	50	9.4	34	8.1	0.486 (1)	0.486
Axis II Primary Diagnoses					
Borderline Personality Disorder	62	6.5	26	4.9	36	8.6	5.258 (1)	0.022
Personality Disorder Not Otherwise Specified	151	15.8	70	13.1	81	19.2	6.648 (1)	0.010
Other Personality Disorder	27	2.8	17	3.2	6	1.4	3.097 (1)	0.078
Diagnosis Deferred	115	12.0	75	14.0	40	9.5	4.589 (1)	0.032
No Diagnosis	401	42.0	231	43.3	169	40.1	0.939 (1)	0.333

Note: The Axis I and Axis II categories reflect major diagnostic categories in the Diagnostic and Statistical Manual of Mental Disorders, Version IV—Text Revision (DSM IV-TR). *df*: degrees of freedom.
